# Supplementary material for: HCV Spread Kinetics Reveal Varying Contributions of Transmission Modes to Infection Dynamics
Source: Viruses. 2021 Jul 6;13(7):1308. doi: 10.3390/v13071308 (PMC8310333; doi:10.3390/v13071308)
Supplement: Supplementary file 1 [file viruses-13-01308-s001.zip › supplemental figures/FigureS1.pdf]

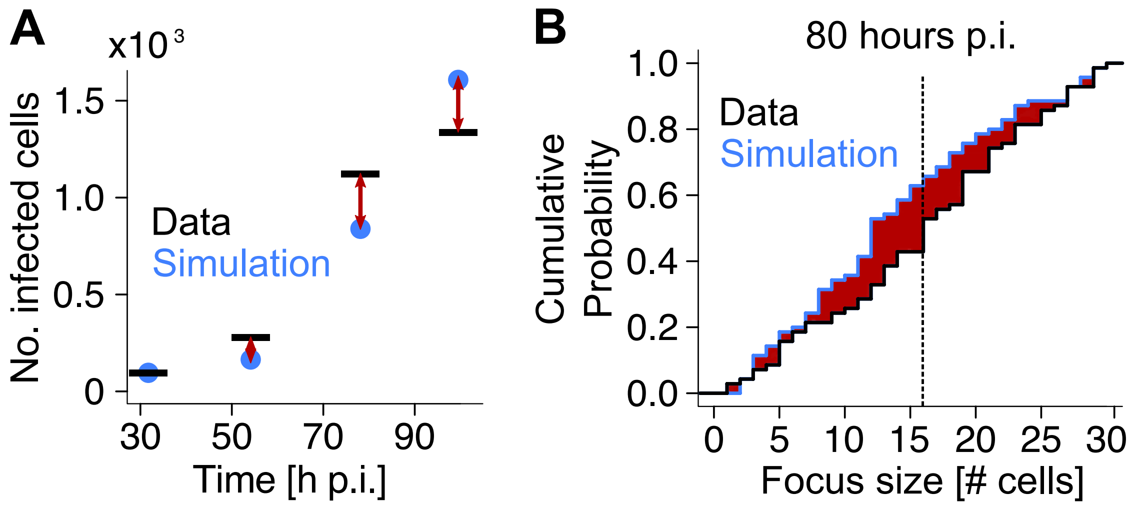

Figure S1: Graph of distance measure used for fitting ABM to in silico or in vitro data. (**A**) Shown is the absolute distance (red arrows) between hypothetical experiment (black lines) and prediction (blue dots). (**B**) The enclosed area of the hypothetical measured (black) and predicted (blue) cumulative density functions of the focus size distribution after 80 hours is indicated with the red shaded area. The dashed line indicates the measured average focus size.
