# Supplementary material for: HCV Spread Kinetics Reveal Varying Contributions of Transmission Modes to Infection Dynamics
Source: Viruses. 2021 Jul 6;13(7):1308. doi: 10.3390/v13071308 (PMC8310333; doi:10.3390/v13071308)
Supplement: Supplementary file 1 [file viruses-13-01308-s001.zip › supplemental figures/FigureS2.pdf]

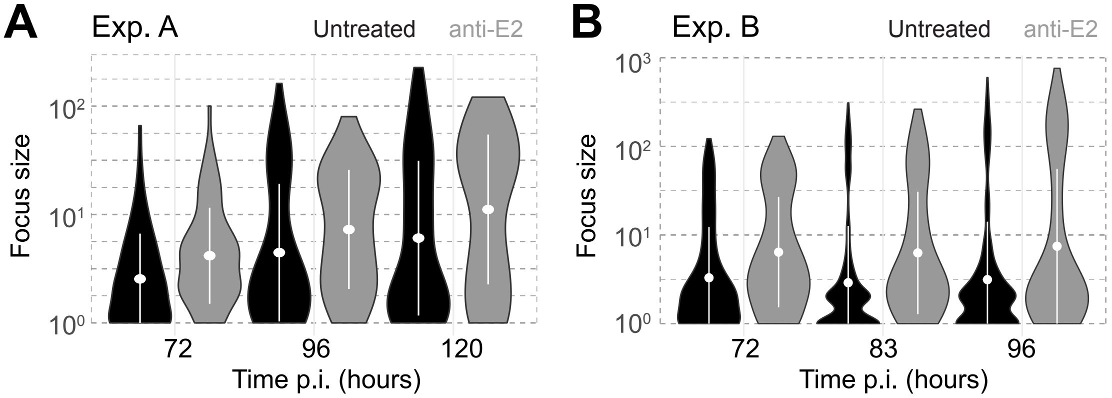

Figure S2: Bi-modal HCV foci size distribution. Foci size distributions for Exp. A (**A**) and Exp. B (**B**) shown as violin plots corresponding to Figure 2B and C in the main text, respectively. Distributions are shown for untreated (black) and anti-E2 (grey) treated cultures with the mean (white dot) and standard deviation (white bar) indicated. The violin plots indicate the bi-modal distribution for anti-E2 treated cultures in Exp. B especially at later time points.
