# Supplementary material for: HCV Spread Kinetics Reveal Varying Contributions of Transmission Modes to Infection Dynamics
Source: Viruses. 2021 Jul 6;13(7):1308. doi: 10.3390/v13071308 (PMC8310333; doi:10.3390/v13071308)
Supplement: Supplementary file 1 [file viruses-13-01308-s001.zip › supplemental figures/FigureS4.pdf]

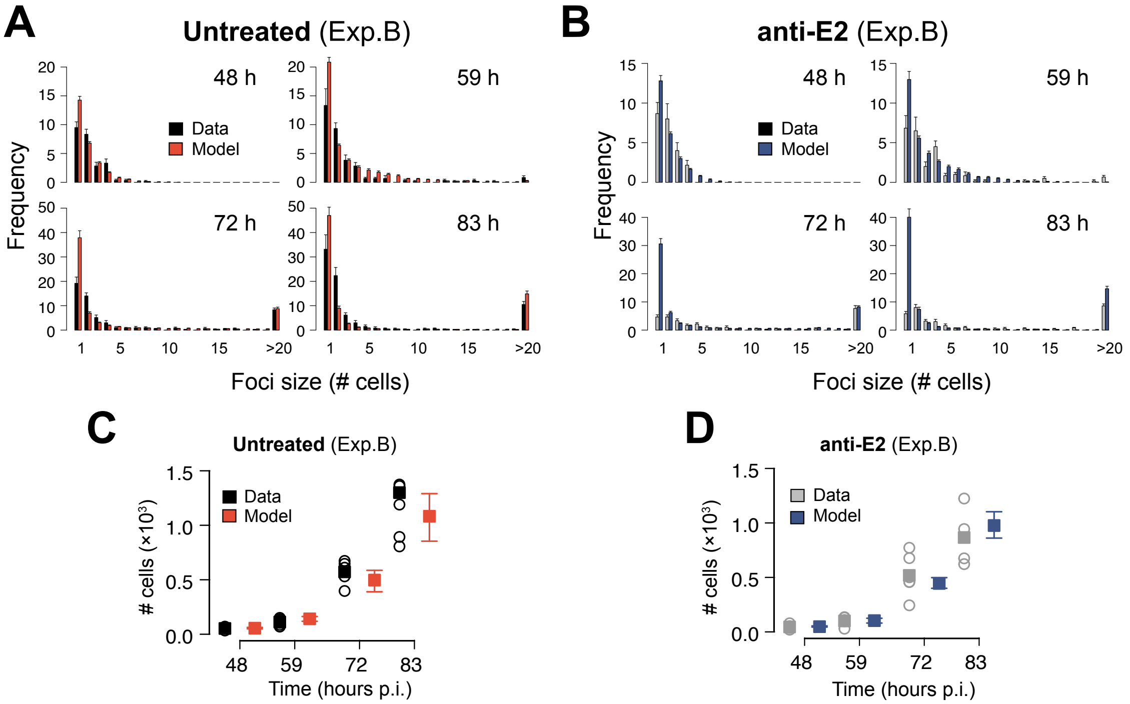

Figure S4: Model predictions for Exp. B. (**A**,**B**) Measured (black/grey) and predicted (red/blue) focus size distributions for Exp. B without (**A**) and with (**B**) administration of anti-E2 at 48, 59, 72 and 83 hours p.i. and calculated across 5 replicates according to the experimental conditions. Predictions are based on an exemplary parameterization of the model as obtained by the fitting procedure. (**C**,**D**) Predicted (red/blue) and measured (black/grey) average number of infected cells in untreated (**C**) and anti-E2 treated (**D**) wells calculated across 5-6 replicates.
