# Supplementary material for: HCV Spread Kinetics Reveal Varying Contributions of Transmission Modes to Infection Dynamics
Source: Viruses. 2021 Jul 6;13(7):1308. doi: 10.3390/v13071308 (PMC8310333; doi:10.3390/v13071308)
Supplement: Supplementary file 1 [file viruses-13-01308-s001.zip › supplemental figures/FigureS5.pdf]

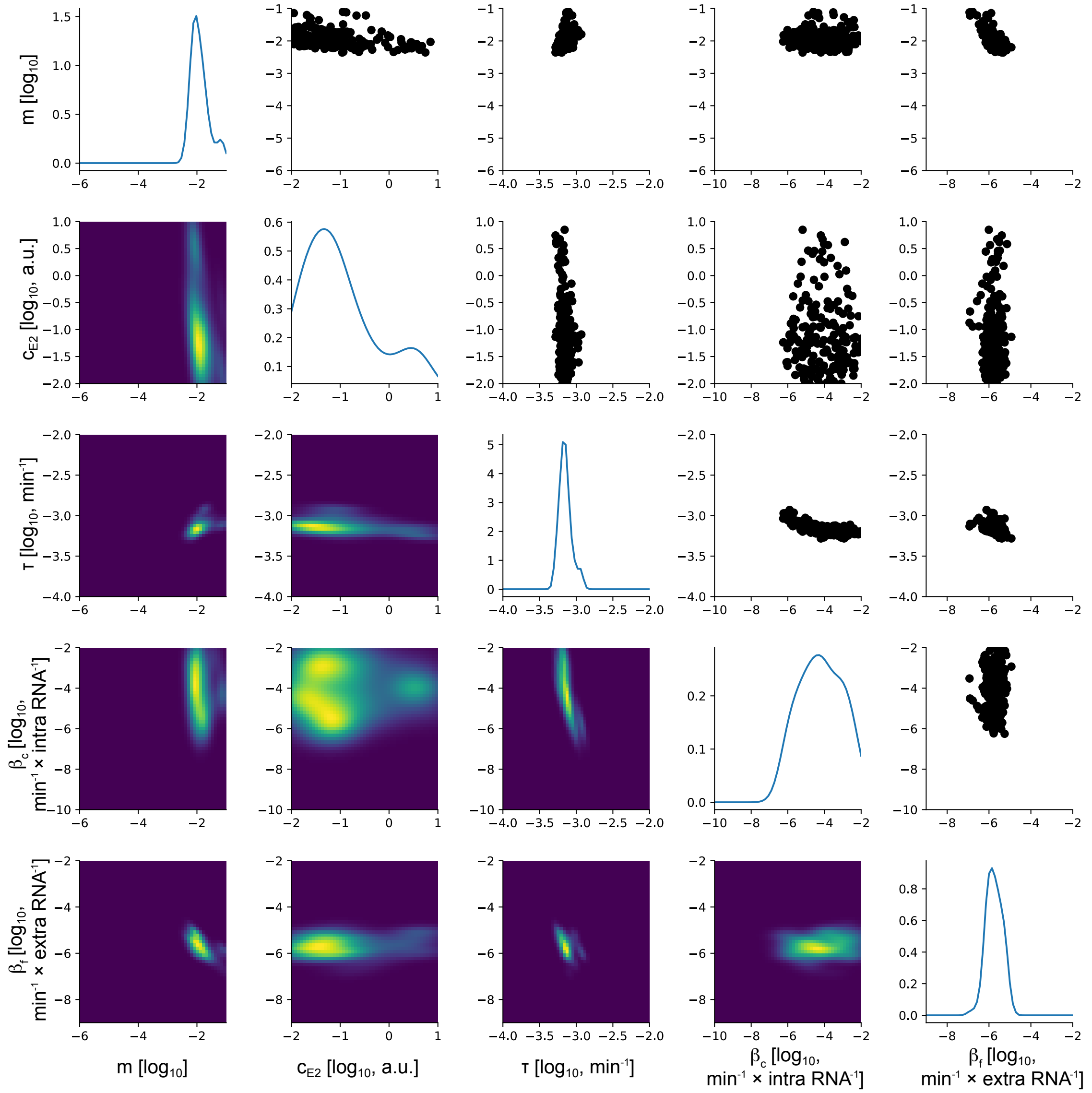

Figure S5: Estimated kernel densities of fitted parameters to in vitro data obtained from Exp. A. Each particle within pyABC used 2 individual ABM-simulations to calculate average infection dynamics according to the experimental conditions. Results shown were obtained after 15 pyABC generations. Individual rows and columns represent the effective diffusion rate of extracellular virus indicated by the coupling coefficient m (1st row/column), the usage rate of anti-E2 c_{E2} (2nd row/column), the cell-to-cell infectivity parameter τ (3rd row/column), and the cell-to-cell (4th row/column) and cell-free (5th row/column) scaling factor β_c and β_f, representing the corresponding transmission rates. Panels above the diagonal represent 200 individual parameter combinations (black), which had a distance smaller than 4.46 and below the diagonal the corresponding estimated two-dimensional kernel densities. The kernel density estimates of each parameter are shown on the diagonal.
