# Supplementary material for: HCV Spread Kinetics Reveal Varying Contributions of Transmission Modes to Infection Dynamics
Source: Viruses. 2021 Jul 6;13(7):1308. doi: 10.3390/v13071308 (PMC8310333; doi:10.3390/v13071308)
Supplement: Supplementary file 1 [file viruses-13-01308-s001.zip › supplemental figures/FigureS7.pdf]

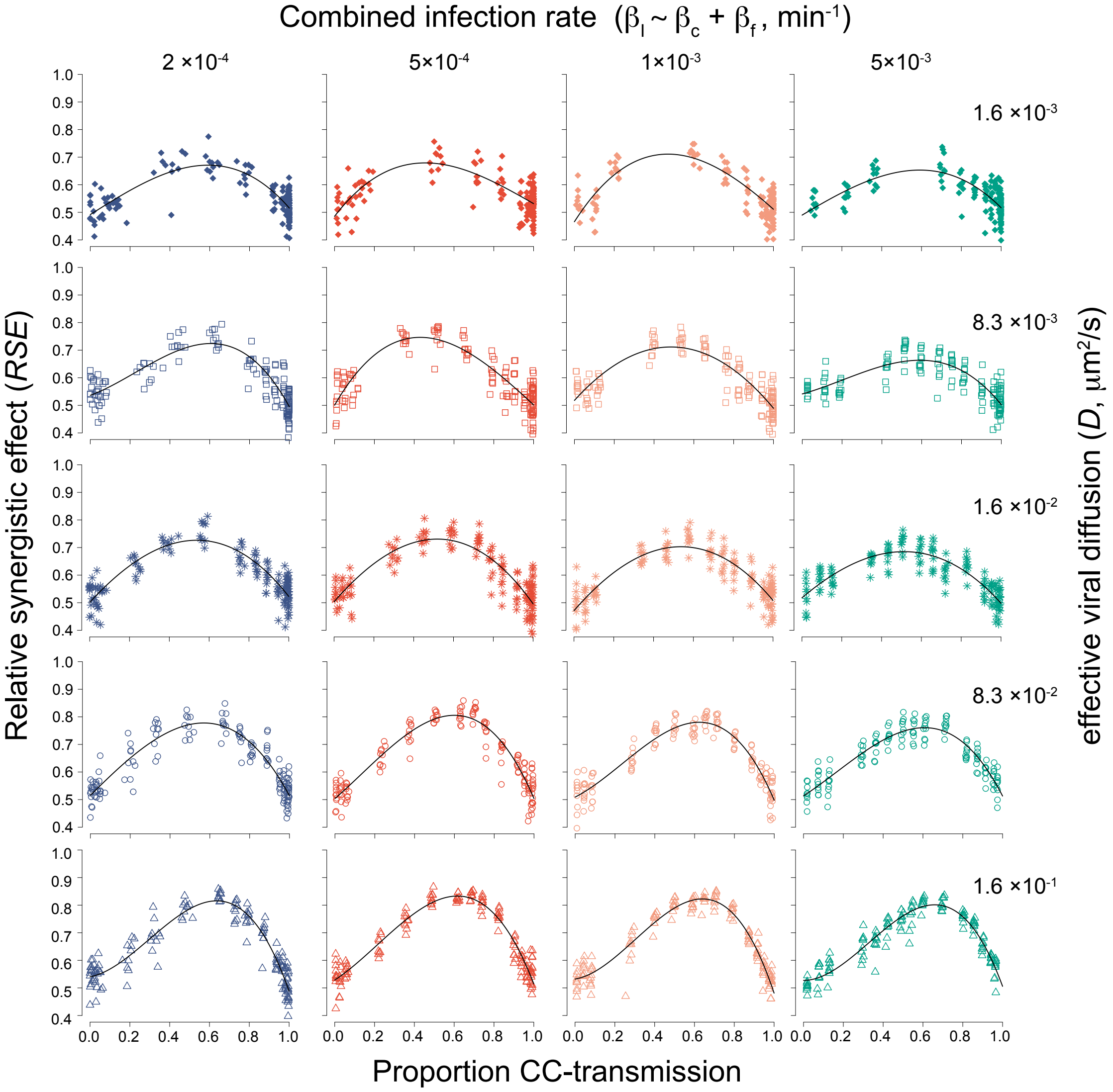

Figure S7: Advantages of combined modes of viral spread. Predicted advantage of combined modes of viral spread indicated by the Relative Synergistic Effect (RSE) of combined viral spread (Expanded set of combinations compared to main manuscript Figure 5). The RSE is shown dependent on the proportion of infections by cell-to-cell transmission for different assumptions of the combined occurrence of infection by both transmission modes and the viral diffusion coefficient. Points indicate the maximal RSE obtained for simulating viral spread in the ABM with different combinations of β_c and β_f making up the combined infection rate β_I. Curves show the result of a polynomial of 3rd degree fitted to the individual data.
