# Supplementary material for: HCV Spread Kinetics Reveal Varying Contributions of Transmission Modes to Infection Dynamics
Source: Viruses. 2021 Jul 6;13(7):1308. doi: 10.3390/v13071308 (PMC8310333; doi:10.3390/v13071308)
Supplement: Supplementary file 1 [file viruses-13-01308-s001.zip › supplemental figures/TableS1.pdf]

Table S1: Parameter estimates for viral lifecycle kinetics using the mathematical model describing intracellular viral replication and viral export (see *Section 2* for a detailed explanation of the individual parameters). Shown are the best fits with numbers in brackets indicating the 95%-confidence intervals obtained by profile likelihood analysis.

Parameter	Description	Unit	Estimate [95%-CI]
R_0	Initial intracellular HCV RNA	RNA $cell^{-1}$	80.1 [42.2, 192]
V_0	Initial residual extracellular HCV RNA	$\times 10^3$ RNA $cell^{-1}$	5.91 [3.66, 10.5]
λ	Intracellular RNA production rate	h^{-1}	0.24 [0.19, 0.28]
ρ	Viral export rate	$\times 10^{-3}$ h^{-1}	2.10 [1.24, 3.47]
R_C	Carrying capacity of intracellular RNA	$\times 10^4$ RNA $cell^{-1}$	7.73 [6.30, 9.76]
c	Viral loss rate	$\times 10^{-2}$ h^{-1}	7.58 [4.88, 11.0]
